# Dihydroflavonol 4-Reductase Genes from *Freesia hybrida* Play Important and Partially Overlapping Roles in the Biosynthesis of Flavonoids

**DOI:** 10.3389/fpls.2017.00428

**Published:** 2017-03-28

**Authors:** Yueqing Li, Xingxue Liu, Xinquan Cai, Xiaotong Shan, Ruifang Gao, Song Yang, Taotao Han, Shucai Wang, Li Wang, Xiang Gao

**Affiliations:** Key Laboratory of Molecular Epigenetics of MOE, Institute of Genetics and Cytology, Northeast Normal UniversityChangchun, China

**Keywords:** *Freesia hybrida*, dihydroflavonol-4-reductase, substrate specificity, transcriptional regulation, functional divergence, cinnamoyl-CoA reductase

## Abstract

Dihydroflavonol-4-reductase (DFR) is a key enzyme in the reduction of dihydroflavonols to leucoanthocyanidins in both anthocyanin biosynthesis and proanthocyanidin accumulation. In many plant species, it is encoded by a gene family, however, how the different copies evolve either to function in different tissues or at different times or to specialize in the use of different but related substrates needs to be further investigated, especially in monocot plants. In this study, a total of eight putative *DFR*-like genes were firstly cloned from *Freesia hybrida*. Phylogenetic analysis showed that they were classified into different branches, and FhDFR1, FhDFR2, and FhDFR3 were clustered into DFR subgroup, whereas others fell into the group with cinnamoyl-CoA reductase (CCR) proteins. Then, the functions of the three *FhDFR* genes were further characterized. Different spatio-temporal transcription patterns and levels were observed, indicating that the duplicated *FhDFR* genes might function divergently. After introducing them into *Arabidopsis dfr* (*tt3-1)* mutant plants, partial complementation of the loss of cyanidin derivative synthesis was observed, implying that FhDFRs could convert dihydroquercetin to leucocyanidin *in planta*. Biochemical assays also showed that FhDFR1, FhDFR2, and FhDFR3 could utilize dihydromyricetin to generate leucodelphinidin, while FhDFR2 could also catalyze the formation of leucocyanidin from dihydrocyanidin. On the contrary, neither transgenic nor biochemical analysis demonstrated that FhDFR proteins could reduce dihydrokaempferol to leucopelargonidin. These results were consistent with the freesia flower anthocyanin profiles, among which delphinidin derivatives were predominant, with minor quantities of cyanidin derivatives and undetectable pelargonidin derivatives. Thus, it can be deduced that substrate specificities of DFRs were the determinant for the categories of anthocyanins aglycons accumulated in *F. hybrida*. Furthermore, we also found that the divergence of the expression patterns for *FhDFR* genes might be controlled at transcriptional level, as the expression of *FhDFR1/FhDFR2* and *FhDFR3* was controlled by a potential MBW regulatory complex with different activation efficiencies. Therefore, it can be concluded that the *DFR*-like genes from *F. hybrida* have diverged during evolution to play partially overlapping roles in the flavonoid biosynthesis, and the results will contribute to the study of evolution of *DFR* gene families in angiosperms, especially for monocot plants.

## Introduction

While there is a range of colors found in plants, the predominant color is green. Pigments in plants have several roles, e.g., photosynthesis, signaling, defense or heat exchange. In order to stand out from the predominant green colors of leaves and stems, plants have flowers (and fruits) with many colors and sometimes multiple color patterns (Miller et al., 2011), which are evloved to attract pollinators as visual signals. Floral pigments mainly include carotenoids, betalains, and flavonoids (Tanaka et al., 2008). In most plant species, flower coloration is primarily caused by flavonoids, and the flavonoid family encompasses at least 6000 molecules, chiefly divided into phlobaphenes, aurones, isoflavonoids, flavones, flavonols, flavanols, and anthocyanins (Hichri et al., 2011). Among them, anthocyanins are the most common pigments found in flowers and fruits (Tanaka et al., 2008; Morita et al., 2014) and, thus, are of particular importance.

**FIGURE 1:**
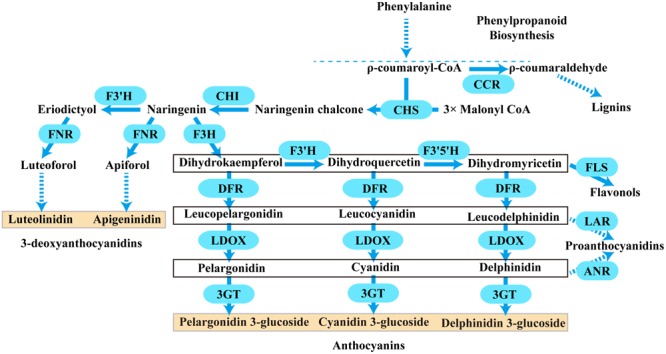
**A schematic diagram of the anthocyanins, 3-deoxyanthocyanidins, lignins, flavonols, and proanthocyanidins biosynthetic pathways in plants.** Bold arrow indicated one-step process. Dotted arrow indicated multi-step catalytic reaction. CCR, cinnamoyl CoA reductase; CHS, chalcone synthase; CHI, chalcone isomerase; F3H, flavanone 3- hydroxylase; F3′H, flavonoid 3′- hydroxylase; F3′5′H, flavonoid 3′,5′- hydroxylase; DFR, dihydroflavonol 4- reductase; LDOX, leucoanthocyanidin di-oxygenase; 3GT, flavonoid 3-*O*-glycosyltransferase; FNR, flavanone 4-reductase; FLS, flavonol synthase; LAR, leucoanthocyanidin reductase; ANR, anthocyanidin reductase.

Anthocyanins are a major class of flavonoids showing bright coloration ranging from blue to orange. Actually, two different types of anthocyanins, 3-hydroxyanthocyanins and 3-deoxyanthocyanins, can be formed in plants (Styles and Ceska, 1975; Halbwirth et al., 2003; Kawahigashi et al., 2016). In contrast to the rare 3-deoxyanthocyanins which have been found only in a few plant species, the ubiquitous 3-hydroxyanthocyanins distribute widely in nature. Thus, the common anthocyanins usually refer to the widely existing 3-hydroxyanthocyanins. Over the past few decades, the biosynthetic pathway of anthocyanins has been well established in plants. They are derived from phenylalanine via the general phenylpropanoid pathway (**Figure 1**). Actually, the general phenylpropanoid pathway also provides precursors for several branches leading to thousands of compounds, for example, lignins could be synthesized from ρ-coumaroyl-CoA by several enzymes containing cinnamoyl-CoA reductase (CCR).

Typically, two classes of genes are confirmed to be involved in the flavonoid pathway: structural genes encoding enzymes that directly participate in the formation of flavonoids, and regulatory genes that control the expression of the structural genes. As shown in **Figure 1**, one molecule of ρ-coumaroyl-CoA and three molecules of malonyl-CoA are catalyzed by chalcone synthase (CHS) to generate naringenin chalcone, which is isomerized to naringenin by chalcone isomerase (CHI). Then, flavanone 3-hydroxylase (F3H) catalyzes the hydroxylation of naringenin to produce dihydrokaempferol (DHK, one hydroxyl group), which is further hydroxylated at the B-ring to form dihydroquercetin (DHQ, two hydroxyl groups) and dihydromyricetin (DHM, 3 hydroxyl groups) by F3′H and F3′5′H, respectively. These genes are usually regarded as early biosynthetic genes (EBGs) in flavonoid biosynthetic pathway. Subsequently, dihydroflavonol 4-reductase (DFR) catalyzes dihydroflavonols to leucoanthocyanidins, which are then converted to stable anthocyanins by leucoanthocyanidin di-oxygenase (LDOX) and flavonoid 3-O-glucosyltransferase (3GT) (Holton and Cornish, 1995; Forkmann and Martens, 2001; Jaakola, 2013). And these genes are designated as late biosynthetic genes (LBGs). Comparably, the 3-deoxyanthocyanidin synthesis pathway shared the same early steps, which are consecutively catalyzed by CHS and CHI. Then the naringenin can be converted to apiforol by flavanone 4-reductase (FNR) or eriodictyol by F3′H. Subsequently, eriodictyol can also be catalyzed into luteoforol by FNR (Lo and Nicholson, 1998; Shih et al., 2006; Liu et al., 2010). In addition, other flavonoids can also be synthesized by partially overlapping or competing pathways. For example, the substrate dihydroflavonol of DFR can be catalyzed by flavonol synthase (FLS) to produce flavonols, and leucoanthocyanidins that result from DFR can be converted to proanthocyanidin by leucoanthocyanidin reductase (LAR), while anthocyanidins resulting from LDOX can also be converted to another kind of proanthocyanidin by anthocyanidin reductase (ANR) (Davies et al., 2003; Xie et al., 2003; Xie and Dixon, 2005; Martens et al., 2010; Yoshida et al., 2010). Herein, FNR, DFR, CCR, ANR, and LAR are important components of the NADPH-dependent reductase superfamily, which fulfill versatile roles in the biosynthesis of plant secondary metabolites.

Dihydroflavonol-4-reductase is a pivotal oxidoreductase (EC 1.1.1.219) catalyzing the NADPH dependent stereospecific reduction of dihydroflavonols, e.g., dihydrokaempferol, dihydroquercetin and dihydromyricetin, to generate leucopelargonidin (LEUP), leucocyanidin (LEUC), and leucomyricetin (LEUM), respectively (Halbwirth et al., 2006; Petit et al., 2007). The substrate specificity of DFR results in different kinds of anthocyanins, mainly delphinidin derivatives, cyanidin derivatives and pelargonidin derivatives. Mutations in DFR may explain the color transition in some species such as Andean genus *Iochroma* (Des Marais and Rausher, 2008; Smith and Rausher, 2011; Smith et al., 2013). To our knowledge, various *DFR* genes have so far been isolated from a wide range of plant species, such as *Lotus japonicus* (Shimada et al., 2005), *Camellia sinensis* (Singh et al., 2009), *Medicago truncatula* (Xie et al., 2004), *Malus domestica* (Fischer et al., 2003), *Pyrus communis* (Fischer et al., 2003), *Citrus sinensis* (Piero et al., 2006), *Ipomoea batatas Lam* (Wang et al., 2013), *Ginkgo biloba* (Cheng et al., 2013), and *Populus trichocarpa* (Huang et al., 2012). However, most of the genes were isolated from dicot plants and few studies have focused on the functionally divergence of the members in the *DFR* gene family, especially in monocot species.

In addition to the structural genes aforementioned, the regulatory mechanism involved in the flavonoid biosynthesis has also been well characterized in plants. Three distinct transcription factor (TF) gene families, containing R2R3 -MYB, basic helix -loop -helix (bHLH) and WD40 repeats (WDRs), comprise a regulatory protein complex (designated as MBW complex) regulating multiple flavonoid metabolisms. At least six MYBs, i.e., PAP1, PAP2, MYB113, MYB114, TT2, MYB5, and three bHLHs, i.e., TT8, GL3, EGL3, and one WD40 (TTG1) regulating the *DFR* expression in *Arabidopsis thaliana* have been well elucidated (Baudry et al., 2004; Gonzalez et al., 2008; Pires and Dolan, 2009; Petroni and Tonelli, 2011; Patra et al., 2013; Xu et al., 2014, 2015). To date, some common TF components of the MBW complex regulating *DFR* expression have been found in maize, petunia, tobacco, and other angiosperms, especially in dicots (Xue et al., 2011; Jaakola, 2013; Xu et al., 2015).

*Freesia*, a monocotyledonous genus of herbaceous perennial flowering plants in the family Iridaceae, is native to the eastern side of southern Africa, and then widely distributes in the world as a cut flower. The freesia flower colors available include red, pink, yellow, white, blue, lavender, purple, and various bicolors. As a result, it has the potential to be a model system for investigating of flavonoid biosynthesis in monocots, particularly for the flower pigmentation. Our previous studies have confirmed the composition of anthocyanin aglycons, i.e., delphinidin, petunidin, malvinidin, peonidin, and cyanidin, and flavonols, i.e., kaempferol and quercetin derivatives, in *Freesia hybrida* “Red River^®^,” Furthermore, we also found that the accumulation profile for anthocyanins was the opposite of that for flavonols during the flower development process (Sun et al., 2016). In addition, proanthocyanidins were detected (Li et al., 2016) which also showed special accumulation patterns. Therefore, the complicated flavonoid compounds in freesia flowers indicated a sophisticated biosynthetic pathway and transcription regulation network for the flavonoid accumulation. So far, two anthocyanin biosynthetic genes, *Fh3GT1* and *FhCHS1*, as well as two bHLH regulatory genes, *FhGL3L* and *FhTT8L*, were isolated and functionally verified (Sui et al., 2011; Sun et al., 2015, 2016; Li et al., 2016). However, no *DFR*-like genes have been isolated and functionally characterized, which is worthy of further concerns because their particular positions in flavonoid biosynthetic pathway.

In the present study, eight putative *DFR*-like genes were firstly cloned from flowers of a universal cultivar of *F. hybrida*, “Red River^®^,” and only three of them were phylogenetically clustered into the DFR subgroup, designated as FhDFR1, FhDFR2, and FhDFR3, respectively, which were further functionally characterized. Their temporal and spatial expression profiles were detected and potential roles *in planta* were investigated through introducing into *Arabidopsis dfr* (*tt3-1)* mutant plants. Furthermore, biochemical properties of FhDFR proteins were also determined. Results indicated that dihydroflavonol 4-reductases performed the crucial roles in the anthocyanin biosynthesis because of substrate specificities, and their functions were at least partially divergent. As expected, the three *FhDFR* genes might be controlled by the common MBW complex with diverse regulation efficiencies, because *Arabidopsis* leaf protoplasts transient expression analysis demonstrated that the earlier characterized FhGL3L and FhTT8L could regulate the expression of *FhDFR1/FhDFR2* and *FhDFR3* coupled with *Arabidopsis* endogenous MYB-type TF, AtPAP1, and the promoter of *FhDFR3* was activated more extensively. Based on the results aforementioned, a model that elucidated the anthocyanin biosynthesis in *F. hybrida* was proposed. To our knowledge, this is the first report of the identification of dihydroflavonol 4-reductase gene family in *F. hybrida*, and the results will not only provide new insights into the flavonoid biosynthesis in monocot plants but also contribute to the study of evolution of *DFR* gene families in angiosperms.

## Materials and Methods

### Plant Materials and Growth Conditions

“Red River^®^,” a cultivar of *F. hybrida* with red flowers, was grown in sandy loam with pH 6.5–7.2 in the greenhouse at 15°C with 14 h/10 h (light/dark) photoperiod. The soil should be kept moist before flower anthesis. For genes isolation and spatio-temporal expression analysis, diverse samples including flowers of five developmental stages with increasing pigmentation intensities and three vegetative tissues, i.e., root, leaf and scape, five flower tissues, i.e., torus, calyx, petal, stamen, and pistil, were collected for RNA extraction as described in our previous studies (Li et al., 2016). All samples were immediately frozen in liquid nitrogen and kept at -80°C prior to total RNA extraction.

*Arabidopsis* mutant *tt3-1* (ABRC stock number: CS84) used for plant transformation was in the Landsberg-0 (Ler) ecotypic background (Shirley et al., 1992), and the seeds were kept at 4°C in the dark for 3 days before grown in a growth chamber at 22°C with 16 h/8 h (light/dark) photoperiod. About 5-week-old plants with several mature flowers in the main inflorescence were used for transformation. In order to study the flavonoid accumulation and expression levels of exogenous *FhDFR* genes from *F. hybrida*, seeds of wild type, mutant and transgenic plants were surface-sterilized, germinated and cultivated in 1/2 Murashige and Skoog (MS) medium (Murashige and Skoog, 1962) supplemented with 3% w/v sucrose.

### RNA Extraction and cDNA Synthesis

RNA was extracted from different samples of *Freesia* or *Arabidopsis* using OminiPlant RNA Kit (DNase I) (CWBIO) following the manufacturer’s instruction. Before cDNA synthesis, RNA was digested with DNase I. cDNA was synthesized in a final reaction volume of 25 μl from total RNA (1 μg) using OligodT 15 primers together with M-MLV Reverse Transcriptase (Promega) according to the manufacturer’s specifications.

### Gene Cloning and Sequence Analysis

To isolate the candidate *DFR*-like genes, *in situ* TBLASTN screen of freesia transcriptomic database, including transcripts from five flower developmental stages and five flower tissues aforementioned, was conducted using *Iris* × *hollandica* DFR (IhDFR, GenBank accession number: BAF93856.1) as probe bait. Sequences obtained were subjected to manual BLASTX search of National Center for Biotechnology Information (NCBI). In order to obtain all the putative candidate *DFR* genes, we defined the sequence as candidate *DFR* genes if several hits were named as *DFR*-like genes in other plant species (Supplementary Table S1). Specific primers were then designed (Supplementary Table S2) to amplify the full length cDNA sequences according to the predicted cDNA sequences. PCR products of appropriate length were cloned into pGEM-Teasy vector (Promega) and then transformed into *Escherichia coli* JM109 competent cells for sequencing confirmation.

Dihydroflavonol-4-reductase and CCR proteins were retrieved from GenBank for multiple sequence alignment following Clustal Omega algorithm (Sievers et al., 2011). Domains for NADP-binding and substrate-binding were highlighted with different colors. Residues directly influencing the substrate specificity were represented by boxes. For phylogenetic analysis, the full-length amino acid sequences of DFR-like proteins from *F. hybrida* and other NADPH-dependent reductases in other plant species were aligned with the Clustal Omega using default parameters^1^, and then the alignments were subjected to MEGA version 6 (Tamura et al., 2013) to generate a neighbor-joining tree with bootstrapping (1,000 replicates) analysis and handling gaps with complete deletion.

### Quantitative Real-Time PCR Analysis

In order to study the spatial and temporal expression patterns of *FhDFR* genes in *F. hybrida*, specific quantitative real-time PCR primers were designed. Transcript levels were analyzed using SYBR Master Mix (TOYOBO, Japan) and a StepOnePlus Real-Time PCR System (Applied Biosystems, USA). All biological replicates were analyzed in triplicate. PCR parameters were set as previously reported (Li et al., 2016). Briefly, a total volume of 10 μl of reaction mixture containing 5 μl of 2 × Master Mix, 0.5 μM of each primer, and 1 μl cDNA were analyzed using the following cycling conditions: 95°C for 60 s, followed by 40 cycles of 95°C for 5 s and 60°C for 60 s. Real-time PCR reactions were normalized to the Ct values for freesia *18S rRNA*. The relative expression levels of the target genes were calculated using the formula 2^-ΔΔCT^ (Livak and Schmittgen, 2001).

### Plant Transformation

All the three *FhDFR* genes digested by *Bam*H I and *Sac* I were cloned into pBI121 vector harboring the *CaMV* 35S constitutive promoter and confirmed by sequencing. The constructs were then transformed to *Agrobacterium tumefaciens* strain GV3101 using a freeze-thaw method. About 5∼6-week old *Arabidopsis* plants with a few mature flowers on the main stems were transformed through the floral dip method (Clough and Bent, 1998). T1 seeds were selected on 1/2 MS medium containing 50 mg L^-1^ kanamycin and transferred to soil to set T2 seeds. The T2 seeds were then cultured on 1/2 MS medium containing 25 mg L^-1^ kanamycin and 3% sucrose. After 1 week of culture on anthocyanin biosynthetic gene induction media, transgenic lines were subjected to evaluate expression level of exogenous *FhDFR* genes and flavonoid accumulation. The *Arabidopsis actin* gene was used as internal control gene when detecting the *FhDFRs* expression levels in transformed mutant lines (Penninckx et al., 1997).

### Measurement of Flavonol and Anthocyanin Contents in *Arabidopsis*

Total anthocyanin content and the amount of flavonol were determined in both wild type, mutants and transgenic plants according to previously described methods (Sun et al., 2016). Briefly, 1-week-old *Arabidopsis* seedlings cultured on 1/2 MS medium with 3% w/v sucrose were ground in liquid nitrogen and submerged in 1 mL H_2_O:MeOH:HCl (75/24/1v/v/v). Extracts were centrifuged and the supernatant was collected. Chromatographic analysis was carried out on a Shimadzu HPLC system equipped with an autosampler with a 20 μl loop, a LC-6AD HPLC Pump and an ACCHROM XUnion C18 column (250 mm × 4.6 mm, 5 μm). The column was eluted with solvent systems A (5% formic acid in H_2_O) and B (methanol) under the following conditions: 0–10 min, 14–17% B; 10–35 min, 17–23% B; 35–60min, 23–47% B; 60–67 min, 47–14% B; 67–70 min, 14% B with a flow rate of 1 ml min^-1^. Detection was monitored at 520 and 360 nm for anthocyanins and flavonols, respectively.

Qualitative analysis of anthocyanin derivatives were conducted by using high performance liquid chromatography (HPLC)-electrospray ionization (ESI)-tandem mass spectrometry (MS) analysis as described earlier (Sun et al., 2016). Briefly, API2000 mass spectrometer (AB Sciex) and SPD- 20AV UV/VIS Detector (Shimadzu, Kyoto, Japan) were equipped with an ESI source. Ion Trap source parameters in positive mode were as follows: ESI source voltage, 4.5 kV; gas (N2) temperature, 450°C; declustering potential, C80 V; entrance potential, 10 V; and scan range, m/z 100–1000 units. Metabolites were identified by their retention times, mass spectra, and product ion spectra in comparison with the data of authentic standards.

### Heterologous Expression of FhDFR Proteins in *Escherichia coli* and *In vitro* Enzyme Assay

Heterologous expression of *FhDFR* genes, which were determined to restore the phenotype of *Arabidopsis* mutant *tt3-1*, and enzyme assay was carried out following the previously described methods (Sun et al., 2015, 2016). Briefly, *FhDFR* genes were subcloned into the *pET28a* vector and expressed as N-terminal His-tagged proteins. An empty vector and vectors harboring different *FhDFR* cDNAs were used for transformation of *E. coli* strain BL21 (DE3). Then the transformants were pre-cultured at 37°C overnight in LB media containing 50 mg L^-1^ kanamycin. The preculture was then transferred to fresh LB media containing 50 mg L^-1^ kanamycin and cultured at 37°C until an *A_600_* of 0.6 was reached. Recombinant proteins were then induced by adding 0.2 mM isopropyl-b-d-thiogalactopyranoside (IPTG), and the optimal induction condition was 28 h and 15°C for FhDFR1 and FhDFR2, 28 h and 20°C for FhDFR3, respectively. After induction, the cells were harvested by centrifugation, resuspended in phosphate-buffered saline (PBS, pH 7.4), and disrupted by sonication. After centrifugation at 13,225 *g* for 20 min, the supernatant containing crude proteins was then applied to 3 ml PBS-equilibrated Ni Sepharose column (GE Healthcare). The column was then washed to remove non-specifically bound proteins using gradient imidazole in PBS. The purified proteins were eluted from the column using 100 mM imidazole in PBS. Eluted FhDFR proteins were desalted in PBS to remove the imidazole at 4°C. The desalted FhDFR proteins were then concentrated and assessed by SDS-PAGE with Coomassie Brilliant Blue staining (Supplementary Figure S1). After that, their concentrations were detected by NanoDrop 1000 (Thermo scientific) Spectrophotometer before enzymatic assays.

Substrate specificities of FhDFR proteins were carried out as described by Cheng et al. (2013). Shortly, DHK, DHM, and DHQ bought from Sigma were dissolved in methanol at 10 mg/mL. A 500 μl reaction mixture consisting of 370 μL of 100 mM Tris-HCl buffer (pH7.0), 70 μL of 0.5 mg/ml FhDFR enzyme extract, 10 μL of substrate, and 50 μL of 20 mM NADPH was kept at 30°C for 30 min. 20 μL of reaction solution was resolved on an ACCHROM XUnion C18 column. The column was eluted with solvent systems A (1% H_3_PO_4_ in water) and B (methanol) under the following conditions: 0 min, 15% B; 0–20 min, 15–60% B; 20–30min, 60–15% B. Detection was monitored at 280 nm, the maximum absorbance wavelength for most of the substrates and products.

### DNA Extraction and Plasmid DNA Preparation Used in *Arabidopsis* Leaf Protoplast Transfection Assay

DNA was extracted from freesia flowers using NuClean Plant Genomic DNA Kit (CWBIO) according to the manufacturer’s instruction. Promoters of *FhDFR1/FhDFR2* and *FhDFR3* were cloned using Genome Walking Kit (TaKaRa) following the instructions. The -1466 bp of *FhDFR1/FhDFR2* and -1132 bp of *FhDFR3* from the initiation condon “ATG” were amplified as promoters and cloned into *Pst* I and *Sac* I digested *AtDFR-pro:GUS* construct to generate *FhDFR1/FhDFR2-pro:GUS* and *FhDFR3-pro:GUS*, respectively. All the other constructs used for protoplasts transfection have been described previously (Li et al., 2016). All the plasmids were prepared using the EndoFree Plasmid Maxi Kit (CWBIO) following the manufacturer’s instructions.

### Protoplast Isolation, Transfection and GUS Activity Assay

Protoplast isolation, transfection and GUS activity assays were performed as described previously (Wang and Chen, 2014; Zhou et al., 2014). Briefly, 3 to 4-week-old Col wild type *Arabidopsis* rosette leaves were collected and used to isolate protoplasts. *FhDFR1/FhDFR2-pro:GUS* and *FhDFR3-pro:GUS* constructs were transformed with different effecter plasmids into protoplasts. A 10 μg aliquot of each plasmid was used in transfection assays. After 20–22 h incubation at room temperature in the dark, the protoplasts were lysed and incubated with 4-methyl-umbelliferyl-β-D-glucuronide (MUG) assay solution at 37°C for 50 min. GUS activities were measured using a Synergy^TM^ HT microplate reader (BioTEK). The assays were repeated three times with three biological replicates.

## Results

### Isolation and Characterization of *DFR*-Like Genes from *Freesia hybrida*

Amino acid sequence of IhDFR was used as bait probe during *in situ* TBLASTN search of transcriptomic database of *F. hybrida.* Consequently, eight putative sequences encoding Rossmann-fold NAD(P)(+)-binding proteins were isolated and predicted as flavonoid reductases (FRs) genes which might be the *DFR*-like genes in freesia. Among them, three genes were named as *FhDFR1, FhDFR2*, and *FhDFR3*, which were most likely to be bona fide *DFR* genes, because they encoded proteins sharing 65, 66, and 65% identities to *Arabidopsis* DFR, and 79, 79, and 77% identities to *Iris* × *hollandica* DFR, respectively (Supplementary Table S1). In contrast, other five genes might encode CCR-like proteins as the best hits of manual BLASTX search were CCRs from other plant species (Supplementary Table S1). Thus, they were tentatively designated as *FhCCR1, FhCCR2, FhCCR3, FhCCR4*, and *FhCCR5*, respectively.

**FIGURE 2:**
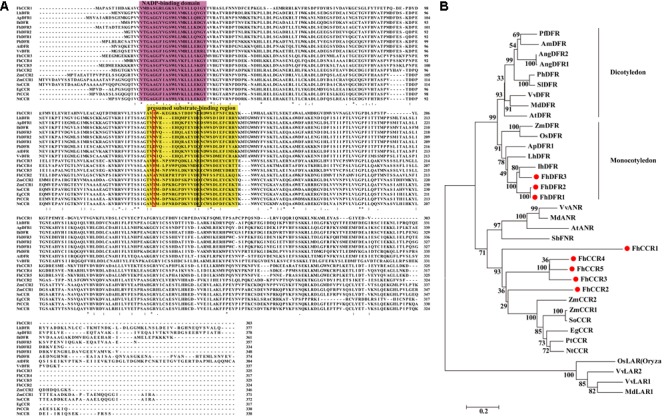
**Alignment of amino acid sequences and phylogenetic analysis of the DFR-like and CCR-like proteins in *Freesia hybrida* with proteins from other species. (A)** Multiple alignment of freesia proteins with DFR and CCR proteins from other species. Numbers indicated the position of the last amino acid in each line of the proteins. ^∗^, identical amino acids; : or ⋅, similar amino acids. The putative NADP binding site and presumed substrate-binding region were shaded in different colors, respectively. Substrate specificity is associated with particularly aa134 and aa145 in the red and black boxes (Johnson et al., 2001). **(B)** Phylogenetic relationships between freesia DFR-like and CCR-like proteins and other NADPH-dependent reductase proteins from other plant species. Phylogenetic tree was constructed using the neighbor-joining method by the MEGA6 software. The reliability of the trees was tested using a bootstrapping method with 1000 replicates. Numbers indicate bootstrap values for 1000 replicates. Freesia proteins were indicated with red circles. The GenBank accession numbers of the protein sequences used are as follows: *Iris* × *hollandica* IhDFR (BAF93856.1); *Agapanthus praecox* ApDFR1 (AB099529.1); *Arabidopsis thaliana* AtDFR (AB033294), AtANR (Q9SEV0.2); *Lilium hybrid* LhDFR (AB058641); *Petunia* × *hybrida* PhDFR (AF233639); *Vitis vinifera* VvDFR (X75964), VvLAR1 (AAZ82410), VvLAR2 (AAZ82411), VvANR (BAD89742); *Angelonia angustifolia* AngDFR1 (KJ817183), AngDFR2 (KF285561); *Zea mays* ZmDFR (Y16040), ZmCCR1 (Y13734), ZmCCR2 (Y15069); *Oryza sativa* OsLAR (CAI56328.1), OsDFR (AB003495); *Perilla frutescens* PfDFR (AB002817); *Malus* domestica MdDFR (AAO39816), MdLAR1 (AAZ79364.1), MdANR (AEL79861.1); *Solanum lycopersicum* SlDFR (CAA79154.1); *Antirrhinum majus* AmDFR (X15536); *Sorghum bicolor* SbFNR (BAU68557.1); *Eucalyptus gunnii* EgCCR (X97433); *Populus trichocarpa* × *P. deltoides* PtCCR (A47097); *Nicotiana tabacum* NtCCR (A47101); *Saccharum officinarum* SoCCR (AJ231134); *Freesia hybrida* FhDFR1 (KU132393), FhDFR2 (KU132389), FhDFR3 (KU132390), FhCCR1 (KU132391), FhCCR2 (KU132392), FhCCR3 (KU132388), FhCCR4 (KU132394), FhCCR5 (KU132395).

Moreover, *FhDFR1* and *FhDFR2* shared the highest nucleotide identity of 95%, in comparison to *FhDFR1, FhDFR2* showed nine exchanges and the last change from “CGA” to “TGA” resulted in a premature stop codon. And this resulted in a substitution of four amino acids and a deletion of 14 amino acids at the C-terminus (**Figure 2A**). Sequence alignment with a number of NADPH-dependent reductases showed that the N-terminus regions of eight *F. hybrida* proteins contained putative NADP-binding region and substrate-binding region which was composed of 26 amino acid residues (Lacombe et al., 1997; Johnson et al., 2001). Moreover, results here also suggested that FhDFR1, FhDFR2, and FhDFR3 were more similar to the identified DFRs in other species, whereas other five proteins tended to be CCRs.

To further investigate the amino acid sequence homology of the eight freesia proteins to other known DFRs and CCRs, as well as other NADPH-dependent reductases such as LAR, ANR, and FNR, a phylogenetic tree was generated by the neighbor-joining method, and the results showed that DFRs from monocots and eudicots were clearly classified into different branches (**Figure 2B**), DFR-like proteins, including FhDFR1, FhDFR2, and FhDFR3, clustered within a subgroup containing proteins from monocot plant species and were most similar to *Iris* × *hollandica* DFR (Katsumoto et al., 2007), indicating that these three DFR-like proteins might participate in the catalyzing of the NADPH-dependent reduction of 2R, 3R-trans-dihydroflavonols to leucoanthocyanidins in the flavonoid biosynthetic pathway. Furthermore, other five proteins from *F. hybrida* clustered independently outside the core DFR branch and fell into a subclade containing CCRs in other species, implying that they might be members of NADPH dependent CCR family.

### The Expression of *FhDFR* Genes Showed Different Correlations with Flavonoid Accumulation in Flower Developmental Process and Plant Tissues

**FIGURE 3:**
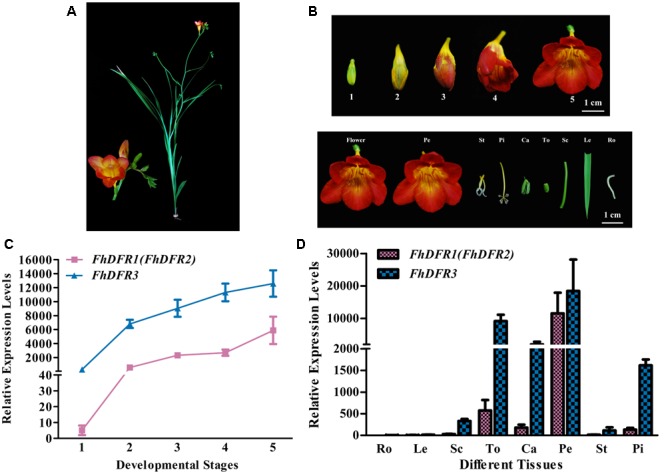
**Expression profiles of *FhDFR* genes in *F. hybrida.* (A)** Phenotypic trait of Red River^®^, a cultivar of *F. hybrida*. **(B)** Five developmental stages of flowers and different tissues. Stage 1 <10 mm long with unpigmented buds; Stage 2 10–20 mm long with slightly pigmented buds; Stage 3 20–30 mm long with pigmented buds; Stage 4 fully pigmented flowers before complete opening; Stage 5 fully opened flowers. Pe, petals; St, stamens; Pi, pistils; Ca, calyxes; To, toruses; Sc, scapes; Le, leaves; Ro, roots. The developmental stages and tissues were selected as described earlier. **(C,D)** Expression profile of *FhDFR* genes in flowers at different developmental stages and in different tissues, respectively. Data represent means ± SD of three biological replicates.

*Freesia hybrida*, as a beautiful perennial herb, sends up a tuft of narrow leaves 10–30 cm long, and a sparsely branched stem 10–40 cm tall bearing a few leaves and a loose one-sided spike of flowers with six tepals (**Figure 3A**). To examine whether the expression patterns of the three potential *FhDFR* genes in flower developmental stages and various tissues coincided with anthocyanin and/or proanthocyanidin accumulation in *F. hybrida*, quantitative real-time PCR was performed to investigate their expression levels temporally and spatially using gene specific primers. It was worth mentioning that as *FhDFR1* and *FhDFR2* showing high homogeneity, no specific primer sets could distinguish the two sequences. *FhDFR1* and *FhDFR2* were evaluated together in expression pattern analysis. Developmental stages of the flower of *F. hybrida* were described earlier as follows (**Figure 3B**): Stage 1, flower buds with non-pigmented tepals; Stage 2, flower buds with pale-red tepals; Stage 3, flower buds with red tepals; Stage 4, flower bud just after anthesis; and Stage 5, fully opened flowers (Sun et al., 2015, 2016; Li et al., 2016). Eight different tissues were also collected as follows: root, leaf, scape, torus, calyx, petal, stamen, and pistil.

Similar expression patterns of *FhDFR* genes were observed among the five flower development stages. The expression of *FhDFR1/FhDFR2* initiated from flower buds with non-pigmented tepals (Stage 1), increased gradually with the development of flowers and peaked when the flowers fully opened (Stage 5), showing an expression pattern synchronous to the anthocyanin accumulation. Moreover, *FhDFR3* was also highly expressed in late stage of flower development process, which might also be involved in the biosynthesis of anthocyanins. Totally, the expression level of *FhDFR3* was significantly higher than *FhDFR1*/*FhDFR2* (**Figure 3C**). As for the expression patterns of the *FhDFR* genes in various plant tissues, quantitative real-time PCR analysis showed that the expression level of *FhDFR3* transcripts was also significantly higher than *FhDFR1*/*FhDFR2* in tested tissues (**Figure 3D**). However, *FhDFR3* was dominantly expressed in the proanthocyanidin accumulated tissues, i.e., torus and calyx and anthocyanin accumulated tissues, petal and pistil (Li et al., 2016). In contrast, the abundant expression of *FhDFR1/FhDFR2* was only observed in petal. Thus, it can be deduced that *FhDFR3* fulfills important roles in the biosynthesis of both anthocyanins and proanthocyanidins in different plant tissues, and *FhDFR1/FhDFR2* might be mainly responsible for the petal pigmentation. However, it is not always clear if their functions are partially or completely redundant given that the expression level of *FhDFR3* was higher than *FhDFR1/FhDFR2* in all the tested tissues (**Figure 3D**). In conclusion, the duplicated *FhDFR* genes might function divergently in the biosynthesis of flavonoids in *F. hybrida*.

### *FhDFR1, FhDFR2*, and *FhDFR3* Could Complement the *Arabidopsis tt3-1* Mutants

**FIGURE 4:**
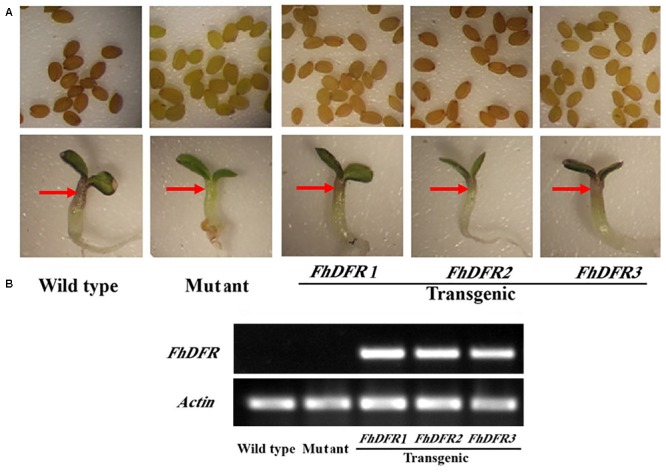
**Complementation of *Arabidopsis tt3-1* mutants overexpressing *FhDFR1, FhDFR2*, and *FhDFR3.* (A)** Phenotype of the wild type (WT, Ler), *tt3-1* mutant and T2 transgenic lines seed coats, cotyledons and hypocotyls. **(B)** Expressional analysis of *FhDFR1, FhDFR2*, and *FhDFR3* by reverse transcription polymerase chain reaction in the wild-type, *tt3* mutant and transgenic lines.

As mentioned above, *FhDFR* genes showed distinct expression patterns and correlations with the accumulation of anthocyanins and proanthocynidins. In order to investigate their potential roles in the biosynthesis of flavonoids *in planta*, the three *FhDFR* genes under the control of *35S* promoter were introduced into the *Arabidopsis* mutant (*tt3-1*), which failed to accumulate anthocyanin pigments in their cotyledon or hypocotyls and brown tannins in their seed coats (**Figure 4A**). After kanamycin selection, seeds of the wild-type, *Arabidopsis* mutant, and T2 transgenic lines were germinated and grown on 1/2 MS medium containing 3% sucrose. Phenotypic observation showed that transgenic plants expressing *FhDFR1, FhDFR2*, and *FhDFR3* genes restored the pigmentation of their seed coats and purple coloration in the cotyledons and hypocotyls (**Figure 4A**), whereas the mutant transformed with the empty vector were green (not shown). The transgenic lines were further confirmed for the presence and expression of exogenous genes through RT-PCR (**Figure 4B**). No amplicons were observed in wild type plants and mutants, whereas amplicons of expected size were observed in transgenic lines.

**FIGURE 5:**
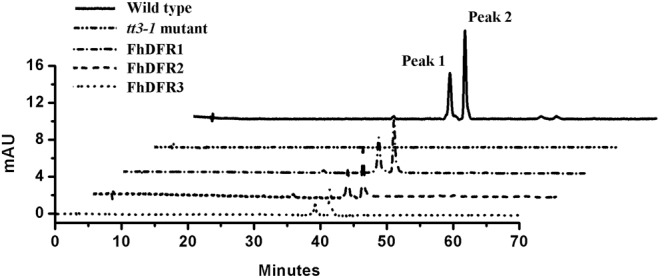
**High performance liquid chromatography analysis of anthocyanins in wild type, *tt3-1* mutant and *Arabidopsis* transgenic seedlings.** The contents of anthocyanins were measured at 520 nm absorbance. Peaks 1 and 2 tended to be cyanidin derivatives, cyanidin 3-*O*-[2-*O*(2-*O*-(sinapoyl)-β-D-xylopyranosyl)-6-*O*-(4-*O*-(β-D-glucopyranosyl)-*p*-coumaroyl)-β-D-glucopyranoside]5-*O*-[6-*O*(malonyl)β-D-glucopyranoside]methyl ester and cyanidin 3-*O*-[2-*O*(2-*O*-(sinapoyl)-β-D-xylopyranosyl)-6-*O*-(4-*O*-(β-D-glucopyranosyl)-*p*-coumaroyl)-β-D-glucopyranoside]5-*O*-[6-*O*(malonyl)β-D-glucopyranoside], respectively.

Furthermore, 1-week-old T2 seedlings cultured on 1/2 MS medium with 3% w/v sucrose were extracted and analyzed by HPLC to determine the amounts of individual anthocyanins and flavonols. The results showed that both wild type and transgenic seedlings (expressing *FhDFR1, FhDFR2, FhDFR3*, respectively) had two primary anthocyanin peaks compared to *tt3-1* mutant seedlings which showed no relative peaks (**Figure 5**). Based on HPLC retention time and MS spectra with authentic compounds, these peaks were identified as cyanidin derivatives (Supplementary Table S3). As for flavonols, no difference in the peak pattern or peak height was observed in wild type plants, mutants or transgenic lines (Supplementary Figure S2). Taken together, these results demonstrated that proteins encoded by *FhDFR1, FhDFR2*, and *FhDFR3* genes could catalyze the NADPH-dependent reduction of dihydroflavonols to leucoanthocyanidins *in planta*.

### Functional Expression in *E. coli* and *In vitro* Catalytic Activities of FhDFR Proteins

**FIGURE 6:**
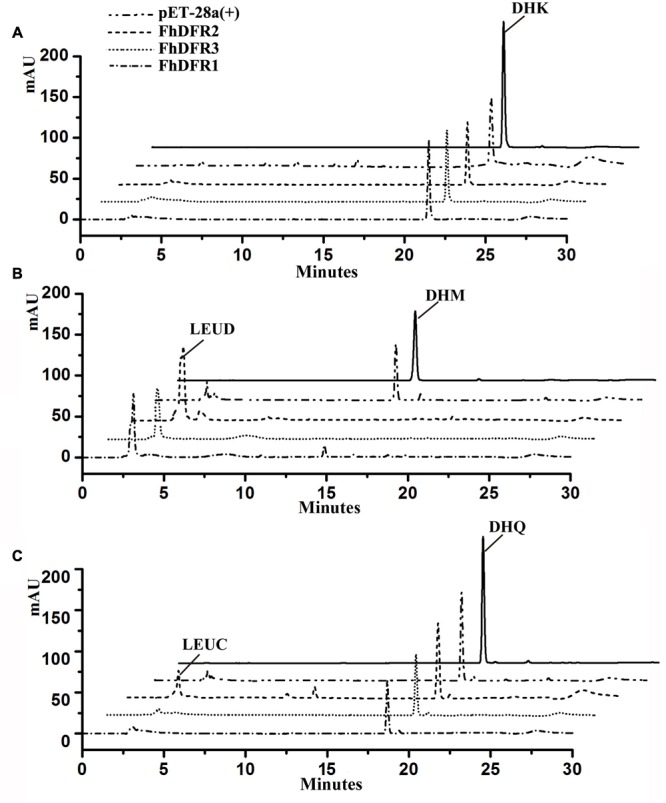
***In vitro* enzyme activity assay of FhDFR1, FhDFR2, and FhDFR3.** Assay mixtures contained NADPH, protein extracts from *E. coli* harboring pET28-*FhDFR2*, pET28-*FhDFR3* and pET28-*FhDFR1*, and DHK **(A)**, DHM **(B)**, and DHQ **(C)** as substrates. Chromatograms were recorded at the UV absorbance wavelength of 280 nm. The identification of the leucocyanidin product was confirmed based on relative retention time and UV spectra.

To further confirm the enzymatic properties of FhDFR1, FhDFR2 and FhDFR3 and their potential roles in the biosynthesis of anthocyanins in *F. hybrida*, substrate specificity studies for FhDFR proteins with a range of substrates were tested, including DHK, DHQ, and DHM. Before catalytic assay, recombinant proteins were prepared and purified. Subsequently, the purified proteins were subjected to the biochemical analysis using DHK, DHQ, or DHM as substrate in the presence of NADPH, respectively, and the reaction products were analyzed by HPLC in comparison to authentic standards, relative retention time and UV spectra (Cheng et al., 2013). As shown in **Figure 6**, formation of leucodelphinidin (LEUD) was observed when using DHM as substrate in the *in vitro* reaction system with FhDFR1, FhDFR2, or FhDFR3 proteins, whereas only FhDFR2 could convert DHQ to LEUC because of relative lower catalytic efficiencies. On the contrary, no LEUP were observed, indicating that FhDFR1, FhDFR2, or FhDFR3 protein might not utilize DHK as substrate. These results revealed that FhDFR1, FhDFR2, and FhDFR3 performed preferentially on DHM *in vitro* as dihydroflavonol 4-reductases.

### *FhDFR1/FhDFR2* and *FhDFR3* Could Be Regulated by AtPAP1 and FhbHLHs

**FIGURE 7:**
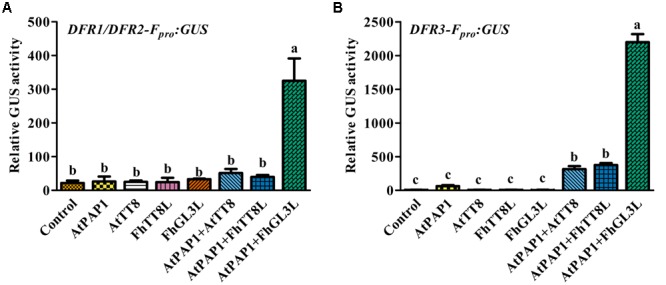
**Promoter activation of *FhDFR*1/*FhDFR*2 and *FhDFR*3 through *Arabidopsis* protoplasts transient assays.** FhGL3L and FhTT8L interacted with AtPAP1 to activate *FhDFR1/FhDFR2* **(A)** and *FhDFR3* **(B)**. Different construct combinations were transfected into *Arabidopsis* protoplasts. The promoter activation abilities were quantified by relative GUS activities. Data represented the mean ± SD of three replicates. Constructs were diagrammed at the bottom of the figure. One-way ANOVA was carried out to compare statistical differences (Ducan, *p* < 0.05).

In our recent study, two IIIf Clade-bHLH regulator genes, *FhGL3L* and *FhTT8L*, participating in anthocyanin and proanthocyanidin accumulation were isolated from *F. hybrida*, and *Arabidopsis* protoplast transfection assay demonstrated that both of them could activate the expression of *AtDFR* in combination with AtPAP1 (Li et al., 2016). In order to verify whether the expression of *FhDFR1, FhDFR2*, and *FhDFR3* genes were also controlled by the MBW complex (AtPAP1-FhbHLHs-AtTTG1) responsible for the anthocyanin biosynthesis, target promoters of *FhDFR1*/*FhDFR2* and *FhDFR3* were firstly isolated. Subsequently, two bHLH regulators, i.e., *FhTT8L* and *FhGL3L* and the MYB regulator *AtPAP1* were independently or co-transfected with the modified *pUC19-GUS* constructs, which contained the target promoters (*FhDFR1/FhDFR2* and *FhDFR3*) driving the expression of the *GUS* reporter gene. As expected, both *FhDFR1/FhDFR2* and *FhDFR3* could be activated by FhGL3L in combination with the MYB protein AtPAP1, whereas FhTT8L could only regulate *FhDFR3* in the presence of AtPAP1. The results here indicated that *FhDFR1/FhDFR2* and *FhDFR3* might be controlled by the MBW complex participated in flavonoid accumulation in *F. hybrida* (**Figure 7**). However, the freesia endogenous MYB regulators still need to be confirmed in the future.

## Discussion

Dihydroflavonol-4-reductase (*DFR*) genes presented strong sequence conservation in plants. In flavonoid biosynthesis, DFR is a member of the short chain dehydrogenase/reductase (SDR) superfamily which contained a highly conserved NADP-binding domain (Johnson et al., 1999, 2001; Martens et al., 2002). In this study, we firstly cloned eight putative homologous cDNA sequences of NADPH-dependent reductase genes in *F. hybrida*. Interestingly, FhDFR1, FhDFR2, and FhDFR3 tended to be DFR-like proteins, whereas other five proteins were more closely related to CCRs through the manual BLASTX search (Supplementary Table S1). Furthermore, FhDFR1 and FhDFR2 showed high similarities in both nucleotide and amino acid sequences. cDNAs synthesized from total RNA of several tissues were used to evaluate the ratio between *FhDFR1* and *FhDFR2*. Primer sets binding to the same site of *FhDFR1* and *FhDFR2* were used and at least 30 clones were sequenced. Results showed the ratio between *FhDFR1* and *FhDFR2* was about 1:1. Bioinformatic analysis found that the proteins encoded by these two genes as well as *FhDFR3* had typical functional domains of DFR proteins, including a conserved NADPH binding motif “VTGAAGFIGSWLIMRLLERGY,” a substrate specificity selective domain and several specific loci of the conservative short-chain dehydrogenase/reductase family (Johnson et al., 1999; Martens et al., 2002). In order to further investigate the potential functions of *DFR*-like genes in *F. hybrida*, we generated a phylogenic tree containing the other characterized NADP-dependent reductases such as DFR, CCR, ANR, LAR, and FNR proteins in other species. As shown in **Figure 2**, both the amino acid alignment and phylogenic tree revealed a higher similarity between FhDFR1, FhDFR2, FhDFR3 and other plants originated and characterized DFRs (**Figure 2**), indicating their potential “DFR-like” catalytic properties. Furthermore, FhDFR1, FhDFR2, and FhDFR3 phylogenetically clustered into the same subgroup with DFRs from other monocot plants. Thus, it can be deduced that the divergence of DFRs most likely occurred after the division of monocots and dicots, and that there might be homologous *DFR* genes in different plants (Liew et al., 1998).

Previous studies demonstrated that substrate specificity of DFR could be influenced by a presumed substrate-binding region composed of 26 amino acid residues, especially the single amino acid residue at about residue 134 (Johnson et al., 2001). Consequently, DFRs could be divided into three types according to differences at this position, i.e., Asn-type DFRs, Asp-type DFRs, and non-Asn/Asp-type DFRs, the amino acid residue of the 134 position was asparagine residue (Asn), aspartic acid (Asp) and neither Asn nor Asp, respectively (Johnson et al., 2001). Generally, Asn-type DFRs could utilize all the three dihydroflavonols, DHK, DHQ, and DHM, as substrates, while Asp-type DFRs could not catalyze DHK efficiently (Forkmann and Ruhnau, 1987; Helariutta et al., 1993; Tanaka et al., 1995; Johnson et al., 1999). However, FhDFR1 and FhDFR2 belonged to Asn-type DFRs, no pelargonidin was detected in flowers of *F. hybrida*, and no LEUP was detected in the *in vitro* catalytic activity assays, which was consistent with ApDFR1 in *Agapanthus praecox* ssp.*orientalis* (Leighton) (Mori et al., 2014). Commonly, Asn-type DFRs are widely distributed in plants, whereas Asp-type DFRs are found in limited plant species that are scattered throughout the eudicots (Shimada et al., 2005). In contrast, FhDFR3, as well as IhDFR from *Iris* × *hollandica*, broke the rule, which belonged to monocotyledonous Asp-type DFRs.

The expression patterns of *FhDFR* genes of *F. hybrida* were tested temporally and spatially. In the development process of flowers, the amount of anthocyanin increased gradually and peaked when flowers fully opened, whereas the proanthocyanidins were constitutively accumulated at a relative low level (Li et al., 2016). *FhDFR1*/*FhDFR2* was parallel well to the anthocyanin accumulation as well as *FhDFR3*, which showed a high expression level at the late stage of the flower development process. In addition, both anthocyanin and proanthocyanidin accumulated more extensively in flower tissues than vegetative tissues, and petal and torus were the dominant tissues for anthocyanin and proanthocyanidin biosynthesis, respectively (Li et al., 2016), which was also synchronous to expression patterns of *FhDFR1/FhDFR2* and *FhDFR3* in these tissues. However, the expression level of *FhDFR3* was significantly higher than *FhDFR1/FhDFR2* genes in all tested tissues, which might imply their partial or complete redundant roles. The spatial and temporal expression characteristics of *FhDFR* genes were found similar in several other species (Nakatsuka et al., 2003; Li et al., 2012; Mori et al., 2014).

In order to investigate the functional divergence of the three *FhDFR* genes in the flavonoid biosynthesis, *FhDFR1, FhDFR2*, and *FhDFR3* were introduced into *Arabidopsis tt3-1* mutants, and the results showed that cyanidin derivatives could be detected in the transgenic plants overexpressing exogenous *FhDFR1, FhDFR2, FhDFR3* genes, indicating that these three genes could utilize DHQ as substrate in *Arabidopsis*. Because the contents of the cyanidin derivatives were significantly lower compared with the wild type plants, it can be deduced that FhDFR1, FhDFR2, FhDFR3 might have relative weaker catalytic efficiency for DHQ in contrast to AtDFR itself. On the other hand, no pelargonidin derivatives were detected in transgenic plants, implying that FhDFR1, FhDFR2, FhDFR3 might be deficient in the catalysis of DHK. As known, there is no DHM accumulated in *Arabidopsis* due to lacking of flavonoid 3′, 5′-hydroxylase (F3′5′H), whether FhDFR1, FhDFR2, FhDFR3 could accept DHM as substrate should be further validated by the examination of their catalytic properties. Based on the *in vitro* biochemical assays, we found that all the three FhDFR proteins aforementioned showed a high catalytic efficiency for DHM, whereas only FhDFR2 was proved to convert DHQ to LEUC, which was not consistent to the results of the *Arabidopsis tt3-1* mutant complementation mentioned above, this contradiction might be ascribed to the lower catalytic efficiency of FhDFR1 and FhDFR3, which was lost during the preparation and purification of the recombinant proteins. In conclusion, FhDFR1, FhDFR2, and FhDFR3 could utilize both DHQ and DHM as substrate, with a higher activity toward DHM than DHQ.

**FIGURE 8:**
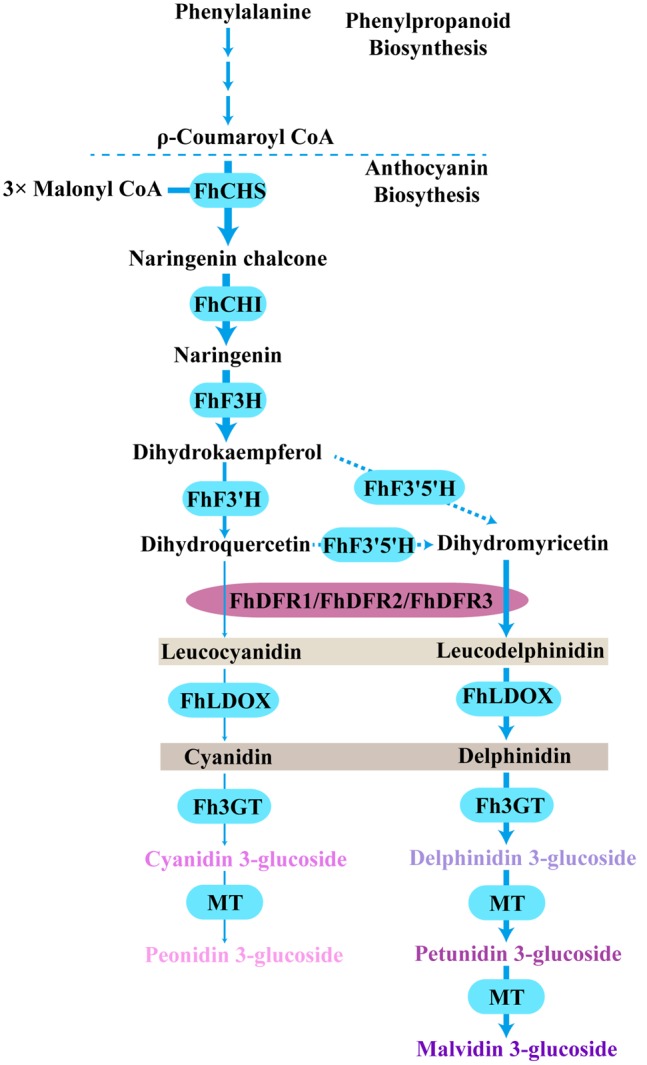
**Proposed pathway leading to anthocyanin biosynthesis in the flowers of *F. hybrida*.** More delphinidin-derived anthocyanins than cyanidin-derived anthocyanins were detected except pelargonidin-derived anthocyanins, FhDFR1, FhDFR2, and FhDFR3 played crucial roles because of their substrate specificities. Bold arrow indicated confirmed process. Dotted arrow indicated uncertain catalytic reaction. CHS, chalcone synthase; CHI, chalcone isomerase; F3H, flavanone 3- hydroxylase; F3′H, flavonoid 3′- hydroxylase; F3′5′H, flavonoid 3′,5′- hydroxylase; DFR, dihydroflavonol 4- reductase; LDOX, leucoanthocyanidin di-oxygenase; 3GT, flavonoid 3-*O*-glycosyltransferase; MT, methyltransferase.

Based on the metabolites found in the flowers, the anthocyanin biosynthetic pathway in freesia was proposed earlier (Sun et al., 2016). All of the six basic anthocyanin aglycons could be synthesized except pelargonidin derivatives, and the most abundant anthocyanins were delphinidin derivatives. On the contrary, flavonol analysis showed predominant kaempferol glycosides and minor quercetin glycosides, whereas myricetin glycosides were undetectable throughout the flower development (Sun et al., 2016). Therefore, as all the precursors of pelargonidin, cyanidin and delphinidin were present in *F. hybrida*, the lack of pelargonidin might be ascribed to the substrate specificity of the FhDFR proteins. In addition, it seemed reasonable to deduce that the lack of myricetin in *F. hybrida* flowers might result from the substrate selectivity of FLS (**Figure 8**). Thus, it was interesting to conclude that FLS and DFR competed for common substrates in order to direct the biosynthesis of flavonols and anthocyanins, respectively, which was also illustrated by Luo et al. (2015) earlier. Actually, several other plant species, such as *Petunia hybrida, Cymbidium hybrida, Angelonia angustifolia, Agapanthus praecox* ssp. *orientalis* (Leighton), have also been found unable to produce pelargonidin derivatives because of the DFR substrate specificities (Mol et al., 1998; Johnson et al., 1999; Gosch et al., 2014; Mori et al., 2014). Therefore, it can be concluded that substrate specificities of FhDFR proteins played crucial role in determination of anthocyanin aglycons in *F. hybrdia*.

The DFRs are key enzymes in flavonoid biosynthesis. Variable *DFR*–like gene numbers were therefore found in various genomes, e.g., a single copy *DFR* is present in *Arabidopsis thaliana*, in which the anthocyanin and proanthocyanidin components seemed to be simple as well, while multicopy *DFR* genes exist in *M. truncatula, L. japonicus, Populus trichocarpa*, and *F. hybrida*, these metabolites showed more complicated patterns and more diverse physiological functions (Østergaard et al., 2001; Xie et al., 2004; Shimada et al., 2005; Huang et al., 2012). Susumu Ohno hypothesized that gene duplication drives the evolution of novel functions, and deduced three kind fates of the duplicated genes: silence, neofunctionalization and subfunctionalization (Epstein, 1971). Based on the phylogenetic position of DFR, ANR, FNR, and CCR, it can be deduced, that they might have diverged from the same ancestral gene after gene or genome duplication during plant evolution. It could also be expected that some DFR isozymes might be specialized to anthocyanin synthesis, proanthocyanidin or other branch pathways in the bioysnthesis of flavonoids. Huang et al. (2012) found that overexpressing *PtrDFR1* in Chinese white poplar (*Populus tomentosa* Carr.) resulted in a higher accumulation of both anthocyanins and condensed tannins, whereas constitutively expressing *PtrDFR2* only improved condensed tannin accumulation. In addition, the different paralogs might be regulated differentially with spatial and temporal manner under exogenous and endogenous cues. In this study, the expression of both *FhDFR1/FhDFR2* and *FhDFR3* was testified to be controlled by a common MBW complex, including MYB and bHLH regulators. However, both *FhDFR1/FhDFR2* and *FhDFR3* could be activated by FhGL3L in combination with the MYB protein AtPAP1, whereas FhTT8L could only regulate *FhDFR3* in the presence of AtPAP1. The endogenous FhMYB regulators still need to be further investigated whether the phenomena simply resulting from the heterogenous AtPAP1 or from the evolutionary divergence between *FhDFR1/FhDFR2* and *FhDFR3*.

## Conclusion

Previous studies showed that gene duplication acted as a driver for plant morphogenetic evolution, and have possibly allowed the adaptation of the enzymes for specialized functions and contributed to the divergence of plant metabolisms (Rensing, 2014). In this study, we found that the duplicated *FhDFR* genes from *F. hybrida* have evolved divergently with different nucleotide sequences and expression patterns. *FhDFR1, FhDFR2*, and *FhDFR3* were involved in the biosynthesis of flavonoinds and determined the components of the anthocyanins in *F. hybrida*. Comparatively speaking, *FhDFR3* might perform more important roles in the biosynthesis of proanthocyanidins. Moreover, they were controlled by a potential MBW complex responsible for anthocyanin biosynthesis. Taken together, the results are not only helpful for future research on DFR evolution and divergence analysis but also useful for manipulating flavonoid biosynthesis in *F. hybrida* as well as in other monocotyledonous ornamental plants.

## Author Contributions

YL, XL, XC, XS, and RG performed most of the experiments. SY and TH helped in seedling planting and sample preparation, SW helped analyze the results, XG designed the experiments, wrote and edited the manuscript. LW helped design the experiments and revise the manuscript. All authors read and approved the final manuscript.

## Conflict of Interest Statement

The authors declare that the research was conducted in the absence of any commercial or financial relationships that could be construed as a potential conflict of interest.

## Footnotes

1 http://www.ebi.ac.uk/Tools/msa/clustalo/

## References

[B1] Baudry A. Heim M. A. Dubreucq B. Caboche M. Weisshaar B. Lepiniec L. (2004). TT2, TT8, and TTG1 synergistically specify the expression of *BANYULS* and proanthocyanidin biosynthesis in *Arabidopsis thaliana*. *Plant J.* 39 366–380. 10.1111/j.1365-313X.2004.02138.x 15255866

[B2] Cheng H. Li L. Cheng S. Cao F. Xu F. Yuan H. (2013). Molecular cloning and characterization of three genes encoding dihydroflavonol-4-reductase from *Ginkgo biloba* in anthocyanin biosynthetic pathway. *PLoS ONE* 8:e72017 10.1371/journal.pone.0072017 PMC3753345 23991027

[B3] Clough S. J. Bent A. F. (1998). Floral dip: a simplified method for *Agrobacterium*-mediated transformation of *Arabidopsis thaliana*. *Plant J.* 16 735–743. 10.1046/j.1365-313x.1998.00343.x 10069079

[B4] Davies K. M. Schwinn K. E. Deroles S. C. Manson D. G. Lewis D. H. Bloor S. J. (2003). Enhancing anthocyanin production by altering competition for substrate between flavonol synthase and dihydroflavonol 4-reductase. *Euphytica* 131 259–268. 10.1023/A:1024018729349

[B5] Des Marais D. L. Rausher M. D. (2008). Escape from adaptive conflict after duplication in an anthocyanin pathway gene. *Nature* 454 762–765. 10.1038/nature07092 18594508

[B6] Epstein C. J. (1971). *Evolution by Gene Duplication.* Berlin: Springer.

[B7] Fischer T. C. Halbwirth H. Meisel B. Stich K. Forkmann G. (2003). Molecular cloning, substrate specificity of the functionally expressed dihydroflavonol 4-reductases from *Malus domestica* and *Pyrus communis* cultivars and the consequences for flavonoid metabolism. *Arch. Biochem. Biophys.* 412 223–230. 10.1016/S0003-9861(03)00013-4 12667486

[B8] Forkmann G. Martens S. (2001). Metabolic engineering and applications of flavonoids. *Curr. Opin. Biotechnol.* 12 155–160. 10.1016/S0958-1669(00)00192-0 11287230

[B9] Forkmann G. Ruhnau B. (1987). Distinct substrate specificity of dihydroflavonol 4-reductase from flowers of *Petunia hybrida*. *Z. Naturforsch.* 42C 1146–1148. 10.1515/znc-1987-9-1026

[B10] Gonzalez A. Zhao M. Leavitt J. M. Lloyd A. M. (2008). Regulation of the anthocyanin biosynthetic pathway by the TTG1/bHLH/Myb transcriptional complex in *Arabidopsis* seedlings. *Plant J.* 53 814–827. 10.1111/j.1365-313X.2007.03373.x 18036197

[B11] Gosch C. Nagesh K. M. Thill J. Miosic S. Plaschil S. Milosevic M. (2014). Isolation of dihydroflavonol 4-reductase cDNA clones from *Angelonia* x *angustifolia* and heterologous expression as GST fusion protein in *Escherichia coli*. *PLoS ONE* 9:e107755 10.1371/journal.pone.0107755 PMC4169556 25238248

[B12] Halbwirth H. Kahl S. Jäger W. Reznicek G. Forkmann G. Stich K. (2006). Synthesis of (C-14)-labeled 5-deoxyflavonoids and their application in the study of dihydroflavonol/leucoanthocyanidin interconversion by dihydroflavonol 4-reductase. *Plant Sci.* 170 587–595. 10.1016/j.plantsci.2005.10.013

[B13] Halbwirth H. Martens S. Wienand U. Forkmann G. Stich K. (2003). Biochemical formation of anthocyanins in silk tissue of *Zea mays*. *Plant Sci.* 164 489–495. 10.1016/S0168-9452(02)00433-8

[B14] Helariutta Y. Elomaa P. Kotilainen M. Seppänen P. Teeri T. H. (1993). Cloning of cDNA coding for dihydroflavonol-4-reductase (DFR) and characterization of *dfr* expression in the corollas of *Gerbera hybrida* var. Regina (Compositae). *Plant Mol. Biol.* 22 183–193. 10.1007/BF00014927 8507822

[B15] Hichri I. Barrieu F. Bogs J. Kappel C. Delrot S. Lauvergeat V. (2011). Recent advances in the transcriptional regulation of the flavonoid biosynthetic pathway. *J. Exp. Bot.* 62 2465–2483. 10.1093/jxb/erq442 21278228

[B16] Holton T. A. Cornish E. C. (1995). Genetics and biochemistry of anthocyanin biosynthesis. *Plant Cell* 7 1071–1083. 10.1105/tpc.7.7.1071 12242398 PMC160913

[B17] Huang Y. Gou J. Jia Z. Yang L. Sun Y. Xiao X. (2012). Molecular cloning and characterization of two genes encoding dihydroflavonol-4-reductase from *Populus trichocarpa*. *PLoS ONE* 7:e30364 10.1371/journal.pone.0030364 PMC3281835 22363429

[B18] Jaakola L. (2013). New insights into the regulation of anthocyanin biosynthesis in fruits. *Trends Plant Sci.* 18 477–483. 10.1016/j.tplants.2013.06.003 23870661

[B19] Johnson E. T. Ryu S. Yi H. Shin B. Cheong H. Choi G. (2001). Alteration of a single amino acid changes the substrate specificity of dihydroflavonol 4-reductase. *Plant J.* 25 325–333. 10.1046/j.1365-313x.2001.00962.x 11208024

[B20] Johnson E. T. Yi H. Shin B. Oh B. J. Cheong H. Choi G. (1999). *Cymbidium hybrida* dihydroflavonol 4-reductase does not efficiently reduce dihydrokaempferol to produce orange pelargonidin-type anthocyanins. *Plant J.* 19 81–85. 10.1046/j.1365-313X.1999.00502.x 10417729

[B21] Katsumoto Y. Fukuchimizutani M. Fukui Y. Brugliera F. Holton T. A. Karan M. (2007). Engineering of the rose flavonoid biosynthetic pathway successfully generated blue-hued flowers accumulating delphinidin. *Plant Cell Physiol.* 48 1589–1600. 10.1093/pcp/pcm131 17925311

[B22] Kawahigashi H. Kasuga S. Sawada Y. Yonemaru J. Ando T. Kanamori H. (2016). The Sorghum gene for leaf color changes upon wounding (*P*) encodes a flavanone 4-reductase in the 3-deoxyanthocyanidin biosynthesis pathway. *G*3 6 1439–1447. 10.1534/g3.115.026104/-/DC1 PMC4856094 26994288

[B23] Lacombe E. Hawkins S. Doorsselaere J. V. Piquemal J. Goffner D. Poeydomenge O. (1997). Cinnamoyl CoA reductase, the first committed enzyme of the lignin branch biosynthetic pathway: cloning, expression and phylogenetic relationships. *Plant J.* 11 429–441. 10.1046/j.1365-313X.1997.11030429.x 9107033

[B24] Li H. Qiu J. Chen F. Lv X. Fu C. Zhao D. (2012). Molecular characterization and expression analysis of dihydroflavonol 4-reductase (DFR) gene in *Saussurea medusa*. *Mol. Biol. Rep.* 39 2991–2999. 10.1007/s11033-011-1061-2 21701830

[B25] Li Y. Shan X. Gao R. Yang S. Wang S. Gao X. (2016). Two IIIf clade-bHLHs from *Freesia hybrida* play divergent roles in flavonoid biosynthesis and trichome formation when ectopically expressed in *Arabidopsis*. *Sci. Rep.* 6:30514 10.1038/srep30514 PMC4964595 27465838

[B26] Liew C. F. Loh C. S. Goh C. J. Lim S. H. (1998). The isolation, molecular characterization and expression of dihydroflavonol 4-reductase cDNA in the orchid, *Bromheadia finlaysoniana*. *Plant Sci.* 135 161–169. 10.1016/s0168-9452(98)00071-5

[B27] Liu H. Du Y. Chu H. Shih C. H. Wong Y. W. Wang M. (2010). Molecular dissection of the pathogen-inducible 3-deoxyanthocyanidin biosynthesis pathway in sorghum. *Plant Cell Physiol.* 51 1173–1185. 10.1093/pcp/pcq080 20529887

[B28] Livak K. J. Schmittgen T. D. (2001). Analysis of relative gene expression data using real-time quantitative PCR and the 2 -ΔΔ C T method. *Methods* 25 402–408. 10.1006/meth.2001.1262 11846609

[B29] Lo S. C. Nicholson R. L. (1998). Reduction of light-induced anthocyanin accumulation in inoculated sorghum mesocotyls. Implications for a compensatory role in the defense response. *Plant Physiol.* 116 979–989. 10.1104/pp.116.3.979 9501130 PMC35099

[B30] Luo P. Ning G. Wang Z. Shen Y. Jin H. Li P. (2015). Disequilibrium of flavonol synthase and dihydroflavonol-4-reductase expression associated tightly to white vs. red color flower formation in plants. *Front. Plant Sci.* 6:1257 10.3389/fpls.2015.01257 PMC4710699 26793227

[B31] Martens S. Preuss A. Matern U. (2010). ChemInform abstract: multifunctional flavonoid dioxygenases: flavonol and anthocyanin biosynthesis in *Arabidopsis thaliana* L. *Phytochemistry* 71 1040–1049. 10.1016/j.phytochem.2010.04.016 20457455

[B32] Martens S. Teeri T. Forkmann G. (2002). Heterologous expression of dihydroflavonol 4-reductases from various plants. *FEBS Lett.* 531 453–458. 10.1016/S0014-5793(02)03583-4 12435592

[B33] Miller R. Owens S. J. Rørslett B. (2011). Plants and colour: flowers and pollination. *Opt. Laser. Technol.* 43 282–294. 10.1016/j.optlastec.2008.12.018

[B34] Mol J. Grotewold E. Koes R. (1998). How gens paint flowers and seeds. *Trends Plant Sci.* 3 212–217. 10.1016/S1360-1385(98)01242-4

[B35] Mori S. Otani M. Kobayashi H. Nakano M. (2014). Isolation and characterization of the dihydroflavonol 4-reductase gene in the monocotyledonous ornamental *Agapanthus praecox* ssp. *orientalis* (Leighton) Leighton. *Sci. Hortic.* 166 1–8. 10.1016/j.scienta.2013.12.009

[B36] Morita Y. Takagi K. Fukuchi-Mizutani M. Ishiguro K. Tanaka Y. Nitasaka E. (2014). A chalcone isomerase-like protein enhances flavonoid production and flower pigmentation. *Plant J.* 78 294–304. 10.1111/tpj.12469 24517863

[B37] Murashige T. Skoog F. (1962). A revised medium for rapid growth and bio assays with tobacco tissue cultures. *Physiol. Plant.* 15 473–497. 10.1111/j.1399-3054.1962.tb08052.x

[B38] Nakatsuka A. Izumi Y. Yamagishi M. (2003). Spatial and temporal expression of chalcone synthase and dihydroflavonol 4-reductase genes in the Asiatic hybrid lily. *Plant Sci.* 165 759–767. 10.1016/S0168-9452(03)00254-1

[B39] Østergaard L. Lauvergeat V. Næsted H. Mattsson O. Mundy J. (2001). Two differentially regulated *Arabidopsis* genes define a new branch of the DFR superfamily. *Plant Sci.* 160 463–472. 10.1016/S0168-9452(00)00407-6 11166433

[B40] Patra B. Schluttenhofer C. Wu Y. Pattanaik S. Yuan L. (2013). Transcriptional regulation of secondary metabolite biosynthesis in plants. *Biochim. Biophys. Acta* 1829 1236–1247. 10.1016/j.bbagrm.2013.09.006 24113224

[B41] Penninckx I. A. Eggermont K. Terras F. R. Thomma B. P. Samblanx G. W. Buchala A. (1997). Pathogen-induced systemic activation of a plant defensin gene in Arabidopsis follows a salicylic acid-independent pathway. *Plant Cell* 8 2309–2323. 10.2307/3870470 PMC161354 8989885

[B42] Petit P. Granier T. D’Estaintot B. L. Manigand C. Bathany K. Schmitter J. M. (2007). Crystal structure of grape dihydroflavonol 4-reductase, a key enzyme in flavonoid biosynthesis. *J. Mol. Biol.* 368 1345–1357. 10.1016/j.jmb.2007.02.088 17395203

[B43] Petroni K. Tonelli C. (2011). Recent advances on the regulation of anthocyanin synthesis in reproductive organs. *Plant Sci.* 181 219–229. 10.1016/j.plantsci.2011.05.009 21763532

[B44] Piero A. R. L. Puglisi I. Petrone G. (2006). Gene characterization, analysis of expression and in vitro synthesis of dihydroflavonol 4-reductase from [*Citrus sinensis* (L.) Osbeck]. *Phytochemistry* 67 684–695. 10.1016/j.phytochem.2006.01.025 16524606

[B45] Pires N. Dolan L. (2009). Origin and diversification of basic-helix-loop-helix proteins in plants. *Mol. Biol. Evol.* 27 862–874. 10.1093/molbev/msp288 19942615 PMC2839125

[B46] Rensing S. A. (2014). Gene duplication as a driver of plant morphogenetic evolution. *Curr. Opin. Plant Biol.* 17 43–48. 10.1016/j.pbi.2013.11.002 24507493

[B47] Shih C.-H. Chu I. K. Yip W. K. Lo C. (2006). Differential expression of two flavonoid 3’-hydroxylase cDNAs involved in biosynthesis of anthocyanin pigments and 3-deoxyanthocyanidin phytoalexins in sorghum. *Plant Cell Physiol.* 47 1412–1419. 10.1093/pcp/pcl003 16943219

[B48] Shimada N. Sasaki R. Sato S. Kaneko T. Tabata S. Aoki T. (2005). A comprehensive analysis of six dihydroflavonol 4-reductases encoded by a gene cluster of the *Lotus japonicus* genome. *J. Exp. Bot.* 56 2573–2585. 10.1093/jxb/eri251 16087700

[B49] Shirley B. W. Hanley S. Goodman H. M. (1992). Effects of ionizing radiation on a plant genome: analysis of two *Arabidopsis transparent testa* mutations. *Plant Cell* 4 333–347. 10.1105/tpc.4.3.333 1354004 PMC160133

[B50] Sievers F. Wilm A. Dineen D. Gibson T. J. Karplus K. Li W. (2011). Fast, scalable generation of high-quality protein multiple sequence alignments using Clustal Omega. *Mol. Syst. Biol.* 7:539 10.1038/msb.2011.75 PMC3261699 21988835

[B51] Singh K. Kumar S. Yadav S. K. Ahuja P. S. (2009). Characterization of dihydroflavonol 4-reductase cDNA in tea [*Camellia sinensis* (L.) O. Kuntze]. *Plant Biotechnol. Rep.* 3 95–101. 10.1007/s11816-008-0079-y

[B52] Smith S. D. Rausher M. D. (2011). Gene loss and parallel evolution contribute to species difference in flower color. *Mol. Biol. Evol.* 28 2799–2810. 10.1093/molbev/msr109 21551271 PMC3203625

[B53] Smith S. D. Wang S. Rausher M. D. (2013). Functional evolution of an anthocyanin pathway enzyme during a flower color transition. *Mol. Biol. Evol.* 30 602–612. 10.1093/molbev/mss255 23155005 PMC3563968

[B54] Styles E. D. Ceska O. C. (1975). Genetic control of 3-hydroxy- and 3-deoxyflavonoids in *Zea mays*. *Phytochemistry* 14 413–415. 10.1016/0031-9422(75)85101-6

[B55] Sui X. Gao X. Ao M. Wang Q. Yang D. Wang M. (2011). cDNA cloning and characterization of UDP-glucose: anthocyanidin 3-O-glucosyltransferase in *Freesia hybrida*. *Plant Cell Rep.* 30 1209–1218. 10.1007/s00299-011-1029-7 21318353

[B56] Sun W. Liang L. Meng X. Li Y. Gao F. Liu X. (2016). Biochemical and molecular characterization of a flavonoid 3-O-glycosyltransferase responsible for anthocyanins and flavonols biosynthesis in *Freesia hybrida*. *Front. Plant Sci.* 7:410 10.3389/fpls.2016.00410 PMC4815329 27064818

[B57] Sun W. Meng X. Liang L. Jiang W. Huang Y. He J. (2015). Molecular and biochemical analysis of chalcone synthase from *Freesia hybrid* in flavonoid biosynthetic pathway. *PLoS ONE* 10:e0119054 10.1371/journal.pone.0119054 PMC4351062 25742495

[B58] Tamura K. Stecher G. Peterson D. Filipski A. Kumar S. (2013). MEGA6: molecular evolutionary genetics analysis version 6.0. *Mol. Biol. Evol.* 30 2725–2729. 10.1093/molbev/mst197 24132122 PMC3840312

[B59] Tanaka Y. Fukui Y. Fukuchi-Mizutani M. Holton T. A. Higgins E. Kusumi T. (1995). Molecular cloning and characterization of *Rosa hybrida* dihydroflavonol 4-reductase gene. *Plant Cell Physiol.* 36 1023–1031. 10.1093/oxfordjournals.pcp.a078844 8528604

[B60] Tanaka Y. Sasaki N. Ohmiya A. (2008). Biosynthesis of plant pigments: anthocyanins, betalains and carotenoids. *Plant J.* 54 733–749. 10.1111/j.1365-313X.2008.03447.x 18476875

[B61] Wang H. Fan W. Li H. Yang J. Huang J. Zhang P. (2013). Functional characterization of Dihydroflavonol-4-reductase in anthocyanin biosynthesis of purple sweet potato underlies the direct evidence of anthocyanins function against abiotic stresses. *PLoS ONE* 8:e78484 10.1371/journal.pone.0078484 PMC3817210 24223813

[B62] Wang S. Chen J. G. (2014). Regulation of cell fate determination by single-repeat R3 MYB transcription factors in Arabidopsis. *Front. Plant Sci.* 5:133 10.3389/fpls.2014.00133 PMC3986520 24782874

[B63] Xie D. Y. Dixon R. A. (2005). Proanthocyanidin biosynthesis–still more questions than answers? *Phytochemistry* 66 2127–2144. 10.1016/j.phytochem.2005.01.008 16153412

[B64] Xie D. Y. Jackson L. A. Cooper J. D. Ferreira D. Paiva N. L. (2004). Molecular and biochemical analysis of two cDNA clones encoding dihydroflavonol-4-reductase from *Medicago truncatula*. *Plant Physiol.* 134 979–994. 10.1104/pp.103.030221 14976232 PMC389921

[B65] Xie D. Y. Sharma S. B. Paiva N. L. Ferreira D. Dixon R. A. (2003). Role of anthocyanidin reductase, encoded by *BANYULS* in plant flavonoid biosynthesis. *Science* 299 396–399. 10.1126/science.1078540 12532018

[B66] Xu W. Dubos C. Lepiniec L. (2015). Transcriptional control of flavonoid biosynthesis by MYB–bHLH–WDR complexes. *Trends Plant Sci.* 20 176–185. 10.1016/j.tplants.2014.12.001 25577424

[B67] Xu W. Grain D. Bobet S. Gourrierec J. L. Thévenin J. Kelemen Z. (2014). Complexity and robustness of the flavonoid transcriptional regulatory network revealed by comprehensive analyses of MYB-bHLH-WDR complexes and their targets in Arabidopsis seed. *New Phytol.* 202 132–144. 10.1111/nph.12620 24299194

[B68] Xue S. Valdez D. Collman P. I. Diamant N. E. (2011). Recent advances on the regulation of anthocyanin synthesis in reproductive organs. *Plant Sci.* 181 219–229. 10.1016/j.plantsci.2011.05.009 21763532

[B69] Yoshida K. Iwasaka R. Shimada N. Ayabe S. Aoki T. Sakuta M. (2010). Transcriptional control of the dihydroflavonol 4-reductase multigene family in *Lotus japonicus*. *J. Plant Res.* 123 801–805. 10.1007/s10265-010-0325-6 20339894

[B70] Zhou L. Zheng K. Wang X. Tian H. Wang X. Wang S. (2014). Control of trichome formation in *Arabidopsis* by poplar single-repeat R3 MYB transcription factors. *Front. Plant Sci.* 5:262 10.3389/fpls.2014.00262 PMC4051193 24959169

