# Supplementary material for: Dihydroflavonol 4-Reductase Genes from Freesia hybrida Play Important and Partially Overlapping Roles in the Biosynthesis of Flavonoids
Source: Front Plant Sci. 2017 Mar 28;8:428. doi: 10.3389/fpls.2017.00428 (PMC5368250; doi:10.3389/fpls.2017.00428)
Supplement: Supplementary file 1 [file Data_Sheet_1.DOC]

Dihydroflavonol 4-reductase Genes from *Freesia hybrida* Play Important and Partially Overlapping Roles in the Biosynthesis of Flavonoids

Yueqing Li ^1^, Xingxue Liu ^1^, Xinquan Cai ^1^, Xiaotong Shan ^1^, Ruifang Gao ^1^, Song Yang ^1^, Taotao Han ^1^, Shucai Wang ^1^, Li Wang^1 *^, Xiang Gao ^1*^

^1^ Key Laboratory of Molecular Epigenetics of MOE and Institute of Genetics & Cytology, Northeast Normal University, Changchun, China

*To whom correspondence should be addressed. E-mail address: gaoxiang424@163.com; wanglee57@163.com

Tel.:+86 431 85099360;


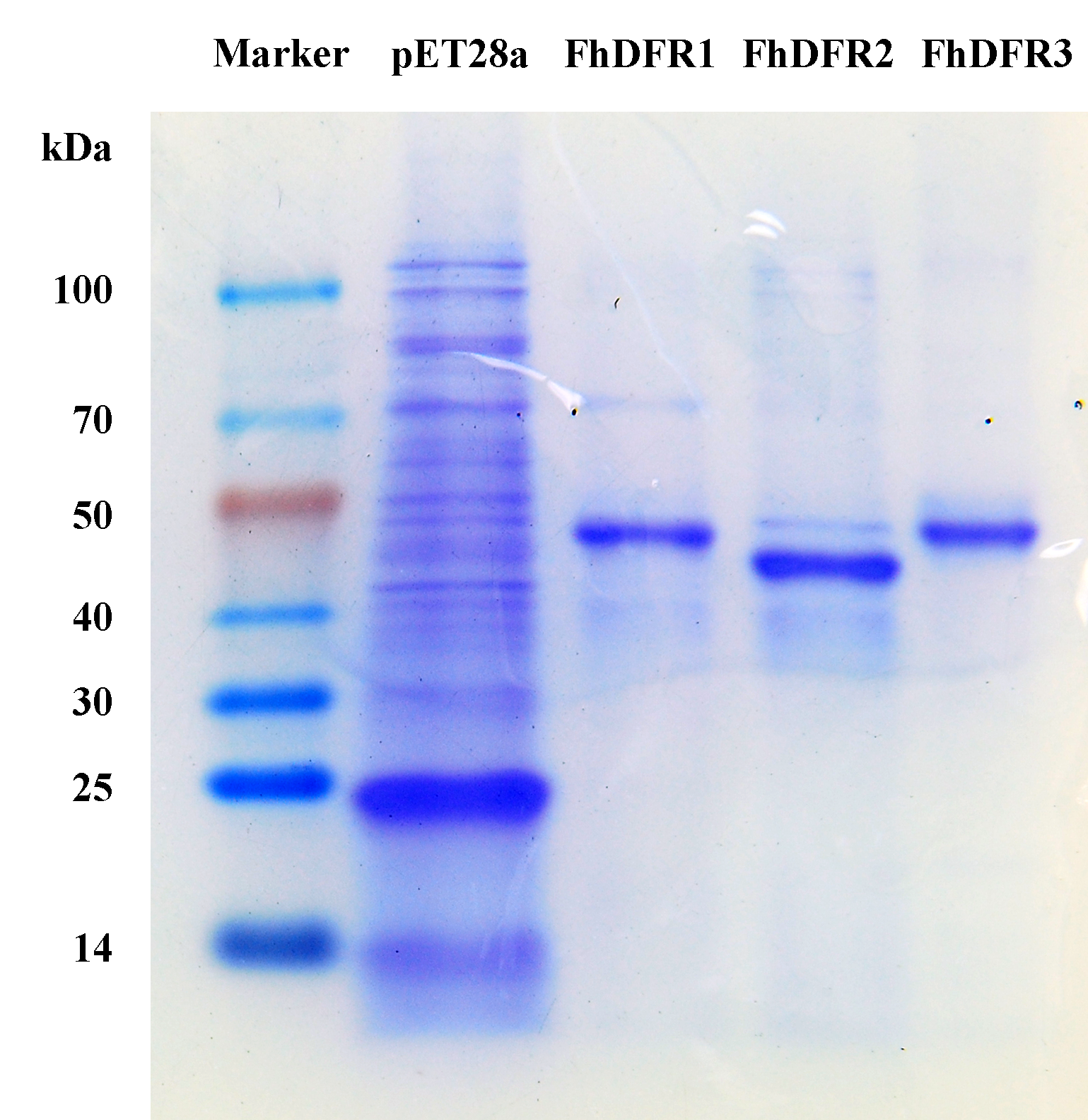


**Figure S1.** SDS-PAGE showing the purified FhDFR1, FhDFR2 and FhDFR3.

2 μg of each purified protein was analyzed by discontinuous polyacrylamide gel (5% stacking gel and 12% running gel) in Tris-glycine buffer. After electrophoresis, the gel was stained using Coomassie Blue G-250 for 1 h and destained using 10% acetic acid and 5% ethanol in a distilled water solution until a clear background was achieved.

**
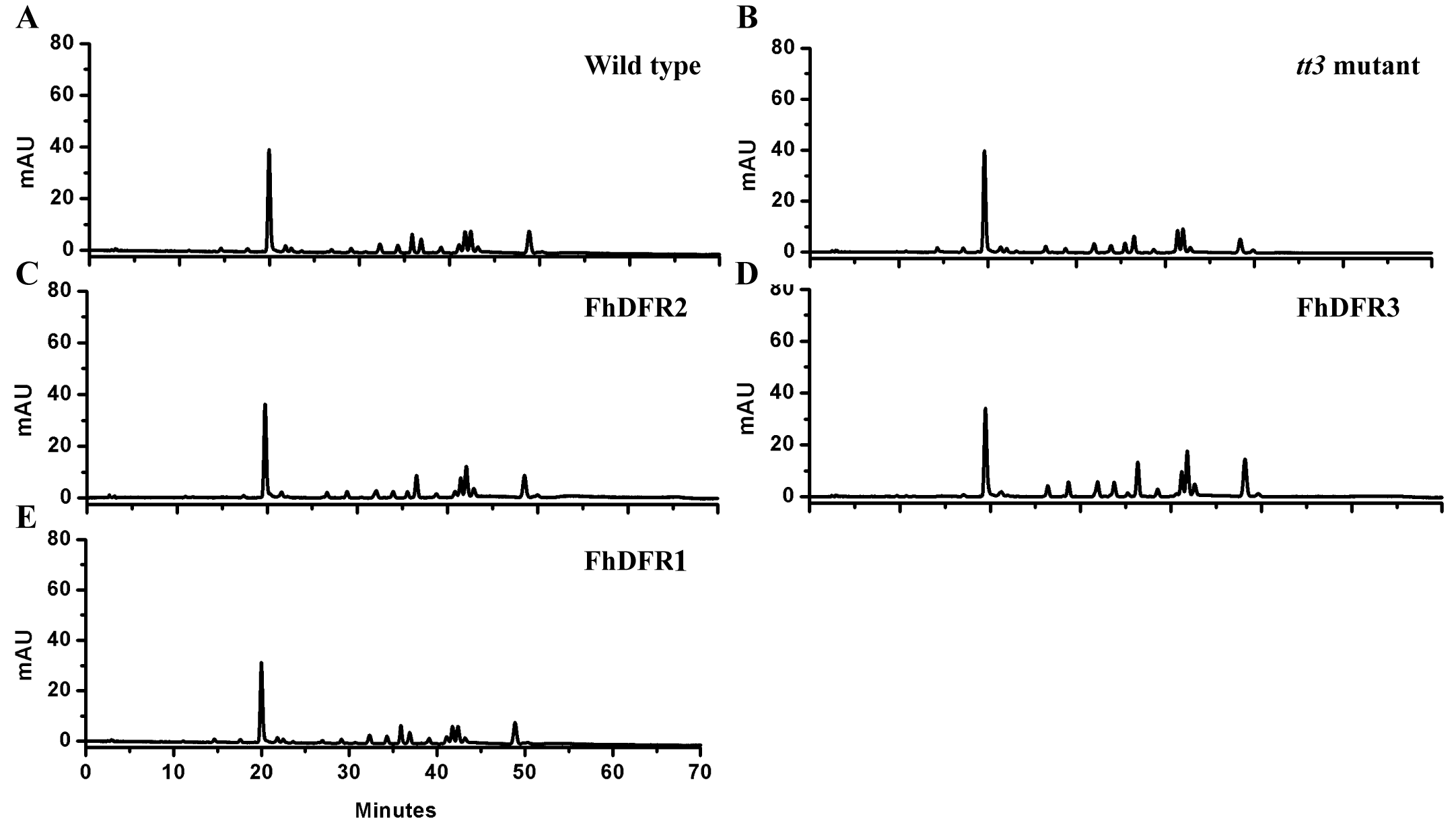
**

**Figure S2.** High performance liquid chromatography analysis of flavonols in wild type, *tt3-1* mutant and *Arabidopsis* transgenic seedlings.

(A) wild type; (B) *tt3-1* mutant; (C) transgenic seedlings overexpressing *FhDFR2*; (D) transgenic seedlings overexpressing *FhDFR3*; (E) transgenic seedlings overexpressing *FhDFR1*. Flavonols were recorded at 360 nm absorbance.

**Table S1. Information of *FhDFR* Genes**

| **Candidate transcripts** | **GenBank**  **number** | **Protein sequence length** | **Top *Arabidopsis* BLAST match** | **Top BLAST match excluding *Arabidopsis*** | **Homology (%)** | **Function**  **prediction** |
| --- | --- | --- | --- | --- | --- | --- |
| FhDFR1 | KU132393 | 348 residues | [NP_199094.1](https://www.ncbi.nlm.nih.gov/protein/15239063?report=genbank&log$=prottop&blast_rank=1&RID=UGY6FN1Y01R) [dihydroflavonol-4-reductase [](https://blast.ncbi.nlm.nih.gov/Blast.cgi#alnHdr_15239063)*Arabidopsis thaliana*] | [BAF93856.1](https://www.ncbi.nlm.nih.gov/protein/160948490?report=genbank&log$=prottop&blast_rank=1&RID=UGXCCDP501R) [dihydroflavonol 4-reductase [](https://blast.ncbi.nlm.nih.gov/Blast.cgi#alnHdr_160948490)*Iris x hollandica*] | 65^a^,79^b^ | Flavonoid reductase (FR) |
| FhDFR2 | KU132389 | 334 residues | [NP_199094.1](https://www.ncbi.nlm.nih.gov/protein/15239063?report=genbank&log$=prottop&blast_rank=1&RID=UGY4FHA601R) [dihydroflavonol-4-reductase [](https://blast.ncbi.nlm.nih.gov/Blast.cgi#alnHdr_15239063)*Arabidopsis thaliana*] | [BAF93856.1](https://www.ncbi.nlm.nih.gov/protein/160948490?report=genbank&log$=prottop&blast_rank=1&RID=UGXA7GVB01R) [dihydroflavonol 4-reductase [](https://blast.ncbi.nlm.nih.gov/Blast.cgi#alnHdr_160948490)*Iris x hollandica*] | 66^a^,79^b^ | Flavonoid reductase (FR) |
| FhDFR3 | KU132390 | 357 residues | [NP_199094.1](https://www.ncbi.nlm.nih.gov/protein/15239063?report=genbank&log$=prottop&blast_rank=1&RID=UGY536NS01R) [dihydroflavonol-4-reductase [](https://blast.ncbi.nlm.nih.gov/Blast.cgi#alnHdr_15239063)*Arabidopsis thaliana*] | [BAF93856.1](https://www.ncbi.nlm.nih.gov/protein/160948490?report=genbank&log$=prottop&blast_rank=1&RID=UGXAR4Z1014) [dihydroflavonol 4-reductase [](https://blast.ncbi.nlm.nih.gov/Blast.cgi#alnHdr_160948490)*Iris x hollandica*] | 65^a^,77^b^ | Flavonoid reductase (FR) |
| FhCCR1 | KU132391 | 303 residues | [NP_565557.1](https://www.ncbi.nlm.nih.gov/protein/18400407?report=genbank&log$=prottop&blast_rank=1&RID=UGY5PBMU01R) [Rossmann-fold NAD(P)-binding domain-containing protein [](https://blast.ncbi.nlm.nih.gov/Blast.cgi#alnHdr_18400407)*Arabidopsis thaliana*] | [XP_008786341.1](https://www.ncbi.nlm.nih.gov/protein/672125826?report=genbank&log$=prottop&blast_rank=1&RID=UGXBAWPV016) [cinnamoyl-CoA reductase 2-like [](https://blast.ncbi.nlm.nih.gov/Blast.cgi#alnHdr_672125826)*Phoenix dactylifera*] | 45^a^,69^b^ | Dihydroflavonol 4-reductase/ cinnamoyl-CoA reductase |
| FhCCR2 | KU132392 | 324 residues | [AAM65984.1](https://www.ncbi.nlm.nih.gov/protein/21594240?report=genbank&log$=prottop&blast_rank=53&RID=UGXBZVYR014) [cinnamyl-alcohol dehydrogenase-like protein [](https://blast.ncbi.nlm.nih.gov/Blast.cgi#alnHdr_21594240)*Arabidopsis thaliana*] | [XP_010905794.1](https://www.ncbi.nlm.nih.gov/protein/743869362?report=genbank&log$=prottop&blast_rank=1&RID=UGXBZVYR014) [cinnamoyl-CoA reductase 1-like [](https://blast.ncbi.nlm.nih.gov/Blast.cgi#alnHdr_743869362)*Elaeis guineensis*] | 74^a^,82^b^ | Bifunctional dihydroflavonol 4-reductase/flavanone 4-reductase/ cinnamoyl-CoA reductase |
| FhCCR3 | KU132388 | 325 residues | [NP_180917.1](https://www.ncbi.nlm.nih.gov/protein/15226134?report=genbank&log$=prottop&blast_rank=82&RID=UGUMFSW5014) [cinnamoyl-CoA:NADP oxidoreductase-like 1 [](https://blast.ncbi.nlm.nih.gov/Blast.cgi#alnHdr_15226134)*Arabidopsis thaliana*] | [XP_008775468.1](https://www.ncbi.nlm.nih.gov/protein/672191282?report=genbank&log$=prottop&blast_rank=1&RID=UGUMFSW5014) [cinnamoyl-CoA reductase 1-like [](https://blast.ncbi.nlm.nih.gov/Blast.cgi#alnHdr_672191282)*Phoenix dactylifera*] | 60^a^,73^b^ | Bifunctional dihydroflavonol 4-reductase/flavanone 4-reductase/ cinnamoyl-CoA reductase |
| FhCCR4 | KU132394 | 317 residues | NP_180917.1 [cinnamoyl-CoA:NADP oxidoreductase-like 1 [](https://blast.ncbi.nlm.nih.gov/Blast.cgi#alnHdr_15226134)*Arabidopsis thaliana*] | [XP_008775468.1](https://www.ncbi.nlm.nih.gov/protein/672191282?report=genbank&log$=prottop&blast_rank=1&RID=UGXCXRZF01R) [cinnamoyl-CoA reductase 1-like [](https://blast.ncbi.nlm.nih.gov/Blast.cgi#alnHdr_672191282)*Phoenix dactylifera*] | 60^a^,75^b^ | Bifunctional dihydroflavonol 4-reductase/flavanone 4-reductase/ cinnamoyl-CoA reductase |
| FhCCR5 | KU132395 | 325 residues | NP_180917.1 [cinnamoyl-CoA:NADP oxidoreductase-like 1 [](https://blast.ncbi.nlm.nih.gov/Blast.cgi#alnHdr_15226134)*Arabidopsis thaliana*] | [XP_008775468.1](https://www.ncbi.nlm.nih.gov/protein/672191282?report=genbank&log$=prottop&blast_rank=1&RID=UGXDD9BB01R) [cinnamoyl-CoA reductase 1-like [](https://blast.ncbi.nlm.nih.gov/Blast.cgi#alnHdr_672191282)*Phoenix dactylifera*] | 60^a^,71^b^ | Bifunctional dihydroflavonol 4-reductase/flavanone 4-reductase/ cinnamoyl-CoA reductase |

a% Similarity to Arabidopsis.

b% Similarity to other plant sequence

**Table S2: Primers Used in the Study**

|  | | Forward(5'-3') | Reverse(5'-3') |
| --- | --- | --- | --- |
| cDNAs |  |  |  |
|  | FhDFR1 | TAGGAAAGAACGATGGGGACGG | GACCATGAACTACACTTTAACCA |
|  | FhDFR2 | AAAGAACGATGGGGACGGTTGT | CTTATTTCACCTTCCAGCTAGTCT |
|  | FhDFR3 | TGATTAGGATACTATCATCAATGGC | CACTCCAGGGCACTAATCTCAG |
|  | FhCCR1 | TACACACATTCTTTCTATCATGGC | ATGCCAATTTGGTCATGTAGAT |
|  | FhCCR2 | GAGAGCGAAAGCGAGCGAGAGA | TCAAATGCTGGGGAGAGAAGGA |
|  | FhCCR3 | GCGGTGGAAGAGAACTAGAGAC | CCAAATACCAATACCCTGAAAA |
|  | FhCCR4 | TAGCGGTGGGAGAGAAGATGAC | AATCTTACAGCCCAGTAGGTCAG |
|  | FhCCR5 | TGGGGATTCCTATTCAAAGTTA | TACCAAACAGGGCATTTATTCT |
| Generation  of constructs used in *Arabidopsis* transformation |  |  |  |
|  | FhDFR1 | CAAGGATCCATGGGGACGGTTGTCGTGA | CAAGAGCTCCTACACTTTAACCATAGCT |
|  | FhDFR2 | CAAGGATCCATGGGGACGGTTGTCGTGA | CAAGAGCTCTCAGCCATTCTCGACTTTGC |
|  | FhDFR3 | CAAGGATCCATGGCCATTACAGCCGACA | CAAGAGCTCTCAGTTTTCAGCAGCAACT |
| Generation  of constructs used in Heterologous expression in  *Escherichia coli* |  |  |  |
|  | FhDFR1 | CAAGGATCCATGGGGACGGTTGTCGTGA | CAAGAGCTCCACTTTAACCATAGCT |
|  | FhDFR2 | CAAGGATCCATGGGGACGGTTGTCGTGA | CAAGAGCTCGCCATTCTCGACTTTGC |
|  | FhDFR3 | CAAGGATCCATGGCCATTACAGCCGACA | CAAGAGCTCGTTTTCAGCAGCAACT |
| qRT-PCR |  |  |  |
|  | FhDFR1 | CCGCTTGGGATTTCGCACTA | TGATCATACTTGGTGGCATAG |
|  | FhDFR2 | CCGCTTGGGATTTCGCACTA | TGATCATACTTGGTGGCATAG |
|  | FhDFR3 | TGGCAGAGTACGACGAGAAC | CCCACAACTAAGGTAGGAATGAT |
|  |  |  |  |
| Generation  of constructs used in *Arabidopsis* leaf protoplasts transient expression system | FhDFR1/2 | AACTGCAGTCCGGCTAATGCCAAAGGTTGA | CAAGAGCTCGGTCACGACAACCGTCCCCAT |
|  | FhDFR3 | AACTGCAGTTTCCACATCATCCAAACAACAGAA | CAAGAGCTCATTCTCTGTGTCGGCTGTAATGG |

Underlined sequences indicate the cutting sites of restriction enzymes.

Table S3. HPLC-DAD and HPLC-ESI-MS analysis of anthocyanins in acidic MeOH-H_2_O extracts of the wild-type Arabidopsis and FhDFRs over-expressing lines

| **Peak number** | **Identification/tentative identification** | **Retention time (min)** | **λmax (nm)** | **ESI-MS**  **(m/z)** |
| --- | --- | --- | --- | --- |
| 1 | cyanidin 3-O-[2-O（2-O-（sinapoyl）-β-D-xylopyranosyl）-6-O-（4-O-（β-D-glucopyranosyl）-p-coumaroyl）-β-D-glucopyranoside]5-O-[6-O(malonyl)β-D-glucopyranoside]methyl ester | 38.92 | 285/526 | 1358 |
| 2 | cyanidin 3-O-[2-O（2-O-（sinapoyl）-β-D-xylopyranosyl）-6-O-（4-O-（β-D-glucopyranosyl）-p-coumaroyl）-β-D-glucopyranoside]5-O-[6-O(malonyl)β-D-glucopyranoside] | 41.18 | 283/537 | 1343 |
